# Did Vaccination Slow the Spread of Bluetongue in France?

**DOI:** 10.1371/journal.pone.0085444

**Published:** 2014-01-21

**Authors:** Maryline Pioz, Hélène Guis, David Pleydell, Emilie Gay, Didier Calavas, Benoît Durand, Christian Ducrot, Renaud Lancelot

**Affiliations:** 1 Unité Mixte de Recherche Contrôle des Maladies Animales Exotiques et Emergentes, Centre de Coopération Internationale en Recherche Agronomique pour le Développement (CIRAD), Montpellier, France; 2 Unité Mixte de Recherche 1309 Contrôle des Maladies Animales Exotiques et Emergentes, Institut National de la Recherche Agronomique (INRA), Montpellier, France; 3 Unité Mixte de Recherche 1309 Contrôle des Maladies Animales Exotiques et Emergentes, Institut National de la Recherche Agronomique (INRA), Petit-Bourg, Guadeloupe, France; 4 Unité Mixte de Recherche Contrôle des Maladies Animales Exotiques et Emergentes, Centre de Coopération Internationale en Recherche Agronomique pour le Développement (CIRAD), Petit-Bourg, Guadeloupe, France; 5 Unité Epidémiologie, Agence Nationale de Sécurité Sanitaire de l'Alimentation, de l'Environnement et du Travail (ANSES), Lyon, France; 6 Laboratoire Santé Animale, Agence Nationale de Sécurité Sanitaire de l'Alimentation, de l'Environnement et du Travail (ANSES), Maisons-Alfort, France; 7 Unité de Recherche 346 d'Epidémiologie Animale, Institut National de la Recherche Agronomique (INRA), Saint Genès Champanelle, France; The Pirbright Institute, United Kingdom

## Abstract

Vaccination is one of the most efficient ways to control the spread of infectious diseases. Simulations are now widely used to assess how vaccination can limit disease spread as well as mitigate morbidity or mortality in susceptible populations. However, field studies investigating how much vaccines decrease the velocity of epizootic wave-fronts during outbreaks are rare. This study aimed at investigating the effect of vaccination on the propagation of bluetongue, a vector-borne disease of ruminants. We used data from the 2008 bluetongue virus serotype 1 (BTV-1) epizootic of southwest France. As the virus was newly introduced in this area, natural immunity of livestock was absent. This allowed determination of the role of vaccination in changing the velocity of bluetongue spread while accounting for environmental factors that possibly influenced it. The average estimated velocity across the country despite restriction on animal movements was 5.4 km/day, which is very similar to the velocity of spread of the bluetongue virus serotype 8 epizootic in France also estimated in a context of restrictions on animal movements. Vaccination significantly reduced the propagation velocity of BTV-1. In comparison to municipalities with no vaccine coverage, the velocity of BTV-1 spread decreased by 1.7 km/day in municipalities with immunized animals. For the first time, the effect of vaccination has been quantified using data from a real epizootic whilst accounting for environmental factors known to modify the velocity of bluetongue spread. Our findings emphasize the importance of vaccination in limiting disease spread across natural landscape. Finally, environmental factors, specifically those related to vector abundance and activity, were found to be good predictors of the velocity of BTV-1 spread, indicating that these variables need to be adequately accounted for when evaluating the role of vaccination on bluetongue spread.

## Introduction

Bluetongue (BT) is a vector-borne disease of ruminants caused by the bluetongue virus (BTV) and transmitted by *Culicoides* biting midges [1]. BT has emerged in Europe since 1998 [2], [3]. Recently, northwest Europe suffered major economic losses during two BT epizootics: the large-scale BTV serotype 8 (BTV-8) epizootic in 2006–2008 [4], [5] and the more restricted BTV-1 epizootic in 2007–2008 [2]. BTV-1 was first detected in southern Spain in the summer 2007. It subsequently spread northward and the two first French clinical cases were reported in November 2007 in southwest France close to the Spanish border. To stop the further spread of the disease in France a massive vaccination campaign was initiated in March 2008. Moreover, restrictions on farm animal movements were implemented in 2007. However, because of limited availability of vaccine doses, vaccination was prioritized in the four departments neighboring the 2007 cases (Pyrénées-Atlantiques, Hautes-Pyrénées, Gers and Landes) and was implemented later in other areas (Fig. S1). Consequently, the level of vaccination coverage during the 2008 vector activity period varied greatly among the different regions and finally more than 4,200 clinical cases were reported. Vaccination ultimately contributed to stop further disease propagation: 83 BTV-1 outbreaks were reported in continental France in 2009, one in June 2010, and none since. However, disease re-emergence can occur, as shown very recently in Corsica where several BTV-1 clinical cases have been reported in September 2013 [6], probably linked with the 2012 BTV-1 epizootic in Sardinia [7].

Numerous studies have been conducted on BT vaccination, essentially experimental, simulation, and observational studies. Experimental studies, for example vaccine challenge studies, in which animals are vaccinated and subsequently challenged with the corresponding pathogenic virus, are of particular interest to assess vaccine efficacy [8]–[12]. Simulation studies have used vaccine efficacy data to estimate disease spread and economic impact under competing vaccination scenarios [13]–[18]. Finally, observational studies have reported metrics such as the number of cases in relation to vaccination coverage [19]–[22]. These studies have shown that vaccination can play an important role in controlling BT spread, reducing both the number of outbreaks and the morbidity and mortality rates in livestock. However, to date no study has investigated the effect of vaccination on the velocity of spread of real BTV epizootics. Two mechanisms are known to influence BT spread: local propagation and long range (>100 km) dissemination. While active flights of infected *Culicoides* and short-range movements of infected farm animals are responsible for local propagation of the infection, BT long range dissemination can occur through the passive flight of infected *Culicoides* carried by winds as well as long distance movements of infected farm animals. Furthermore, mechanisms of BT diffusion in wildlife pass unnoticed. In this study we are interested in the influence of vaccination on local BT propagation: how much could vaccination slow down the progression of BT epizootics in a real agricultural landscape in the presence of restrictions placed on animal movements? Quantitative answers to this question are currently unavailable despite their great potential importance concerning vaccination campaign optimization in the event of another BT epizootic. Since livestock in southwest France had never been in contact with BTV prior to 2007, natural immunity was absent. This allowed us to quantify the importance of vaccination in changing the velocity of spread of the BTV-1 epizootic while accounting for other factors known to influence velocity [23]. We used a similar approach to a previous study of the French BTV-8 epizootic to estimate the velocity of BTV-1 spread [24] and determine which environmental factors influenced velocities [23]. The aim of this study was to quantify the effect of vaccination on the velocity of BTV-1 spread, while accounting for environmental factors known to influence it.

## Materials and Methods

We used 2008 BTV-1 clinical case records from the French Official Veterinary Services to assess the velocity of BTV-1 spread during the 2008 BTV-1 epizootic in France. A case was defined as a bovine herd or an ovine or goat flock in which BT was clinically suspected and BTV-1 infection later confirmed by serological or virological analyses. Our analysis was performed on a municipality basis (the smallest administrative unit in France). Overall 4,195 BTV-1 clinical cases were reported in 1,649 municipalities. Due to clinical cases with missing date of report we discarded 54 municipalities leaving 1,595 municipalities belonging to 16 departments. Data on French BT cases are available on request to the French Official Veterinary Services (Direction générale de l'alimentation, bureau de la santé animale, email: bsa.sdspa.dgal@agriculture.gouv.fr).

### 1 Velocity of BTV-1 spread estimation

To estimate the velocity of BTV-1 spread we used the method described in details in Pioz *et al.* 2011 [24]: a Trend Surface Analysis (TSA) model combined with a spatial error form of Simultaneous Autoregressive model (SAR_err_). Briefly, TSA uses least squares regression to fit polynomial surfaces to geo-referenced event-time data and is used to study diffusion processes in space and time [25]. It has previously been used to identify the pattern of disease diffusion and assess the velocity of spread of rabies [26]–[28], plague [29] and BTV-8 [24]. This method aims to capture the general direction(s) and speed(s) of disease progression. Here, a polynomial surface was fitted to the set of spatially distributed times of first BTV-1 clinical case detection across the 1,595 municipalities. The geographical coordinates (X, Y) of municipality centroids were translated into (X, Y) coordinates with the origin adjusted to the French area of BTV-1 introduction, *i.e.*, the first municipality which reported a BTV-1 case on November 10^th^ 2007. We combined the TSA model with a spatial error form of a Simultaneous Autoregressive (SAR_err_) model to account for the residual spatial autocorrelation. Velocity was estimated by fitting this model to the dates of the first reported clinical case in each municipality. Centroid coordinates for each municipality were used as covariates for the TSA and for generating neighbourhood lists for the SAR_err_ model. We used a model averaging procedure based on AICc to account for model selection uncertainty and obtain robust estimates of model parameters [30] (see details in Material S1).

### 2 Effect of vaccination on velocity of BTV-1 spread

Quantifying the effects of vaccination on the velocity of BTV-1 spread required the most accurate estimates of velocity. We assumed that the estimated velocity was close to the true velocity if the date of the first case predicted by the TSA-SAR_err_ model was close to the observed one. Consequently, following what had been done in the BTV-8 study [23], we restricted the dataset to the municipalities for which the absolute difference between the observed and TSA-SAR_err_ predicted date of the first clinical case was less than 16 days. We used this threshold as a trade-off between discarding strong outliers whilst keeping most of the variability present in the dataset. We checked that the range and characteristics of the environmental factors (minimum, maximum, 1^st^ and 3^rd^ quartiles, median and mean) in the restricted dataset remained close to those of the full dataset (Material S2).

#### 2.1 Ecological variables

Our previous study of BTV-8 spread in France indicated that the velocity of BT spread could be influenced by environmental factors [23]. Thus, to measure the effect of vaccination on the velocity of BTV-1 spread, we needed to consider the variables that may influence the velocity of BT spread and consequently, tested the same covariates as in the BTV-8 study [23]. These covariates are related to host availability and immunity, vector abundance and activity, and vector-host contact. Hence, sixteen covariates defining five thematic groups of related variables were tested (Table 1). Host availability, vaccination, elevation and landscape-related variables were obtained at the municipality level. Meteorological-related variables were obtained on an 8×8 km square grid through the SAFRAN database supplied by Météo-France [31]. Detailed information on the covariates is provided in Material S3, and we detail here only the vaccination covariate. In order to quantify velocity of spread, we focused on the spread of BTV-1 over newly-contaminated areas and assumed that the movements of infected farm animals had only negligible effects on the velocity of BTV-1 spread due to imposed restrictions on animal movements (see [24] for a discussion). Restrictions on farm animal movements were implemented through the European Commission Regulation No 1266/2007, which defined a restricted zone for BT as a 70-km radius around contaminated farms. Regulations on animal transport prevented any movements from restricted zones to non-restricted zones.

**Table 1:** Description of the 16 environmental explanatory covariates tested to model the velocity of BTV-1 spread in France in 2008.

Covariate	Definition	Resolution	Number of classes for qualitative variables (* for quantitative variables)	Classes							
				a		b		c		d	
				range	n	range	n	range	n	range	n
Elevation	Average elevation of the municipality (in meters above sea level)	municipality	4	4–144	329	145–279	331	280–454	327	455–2044	327
Rain_lag1	Total monthly rainfall the month prior to the 1st clinical case of BTV-1 (in mm)	8×8 km grid	4	7.6–34.9	320	35.0–45.9	331	46.0–58.9	328	59.0–121.0	335
Rain_lag2	Total monthly rainfall two months prior to the 1st clinical case of BTV-1 (in mm)	8×8 km grid	4	6.2–36.9	320	37.0–48.5	330	48.6–68.3	336	69.4–207.0	328
Tmax_lag1	Monthly average of the maximal daily temperature the month prior the 1st clinical case of BTV-1 (in °C)	8×8 km grid	4	13.9–23.6	323	23.7–24.3	331	24.4–25.1	330	25. 2– 28.8	330
Tmax_lag2	Monthly average of the maximal daily temperature two months prior the 1st clinical case of BTV-1 (in °C)	8×8 km grid	4	15.2–23.1	336	23.2–24.7	346	24.8–25.8	316	25.9–29.0	316
DensBeef_ Cattle	Density of beef cattle older than 2 months in 2008 (number per km^2^)	municipality	4	0–3.9	336	4.0–12.9	325	13.0–28.9	325	29.0–208	328
DensDairy_Cattle	Density of dairy cattle older than 2 months in 2008 (number per km^2^)	municipality	4	0	319	0.1–1.2	336	1.3–7.9	327	8–135	332
DensSmall_ Ruminants	Density of sheep and goat older than 6 months in 2008 (number per km^2^)	municipality	4	0–0.9	305	1–4.9	330	5.0–19.9	335	20–916	344
VaccinCoverage	Percentage of animals with complete immunity after vaccination at the date of the 1st clinical case of BTV-1 in the municipality	municipality	2	0	1028	0.01–100	286				
arable-pasture	Edge density between arable land and pastures (in m/ha)	municipality	4	0	750	0.01–0.50	185	0.51–1.49	188	1.50–15.01	191
arable–forest	Edge density between arable land and deciduous and mixed forests (in m/ha)	municipality	4	0	573	0.01–0.99	256	1.00–2.99	243	3.00–18.72	242
forest-pasture	Edge density between deciduous and mixed forests and pastures (in m/ha)	municipality	4	0	445	0.01–1.12	288	1.13–3.99	289	4.00–32.2	292
SIDI	Simpson's Diversity Index	municipality	*	0–0.85	1564						
p_arable	Percentage of arable land in the municipality (in %)	municipality	*	0–100	1564						
p_pasture	Percentage of pastures in the municipality (in %)	municipality	*	0–83	1564						
p_forest	Percentage of deciduous and mixed forests in the municipality (in %)	municipality	*	0–90	1564						

For qualitative variables number of classes, range and number of municipalities in each class are presented, for quantitative variables range is presented. N = 1,314 municipalities.

#### 2.2 Vaccination

A compulsory BTV-1 vaccination scheme was implemented in France from late March to December 2008. Early vaccination, *i.e.,* before the springtime onset of vector activity, was prioritized in areas surrounding the 2007 cases (Fig. S1). Vaccination began later in other areas. The inactivated vaccines ZULVAC®1 Bovis and ZULVAC®1 Ovis were used for cattle and small ruminants, respectively. After the second vaccine dose, time to full protection is 15 days in cattle and 24 days in small ruminants. Vaccine efficacy was assumed to be 100%. Expenses for livestock vaccination were covered by the French Ministry of Agriculture and data on BTV-1 vaccination were provided by FranceAgriMer, which is the organisation that paid the veterinarians who performed vaccination. Vaccine coverage in a municipality was calculated as the proportion of small ruminants and cattle immunized at the date of the first BTV-1 clinical case in the municipality, *i.e.*, the ratio of the number of small ruminants and cattle that reached full protection at the date of the first BTV-1 clinical case, to the sum of the number of small ruminants reported on January 2008 and the number of cattle over 2 months old reported on September 2008. We expected lower velocities in areas with high vaccine coverage. Indeed, the higher the vaccine coverage, the lower the proportion of susceptible hosts, and the lower the proportion of infectious hosts. Thus, vaccination decreased the proportion of infectious vectors and the probability of an infectious midge bite.

#### 2.3 Statistical analysis

To prevent statistical issues associated with multi-collinearity we verified that covariates were not highly correlated. For this purpose, we computed correlations among all the covariates (Table S1). Since covariates were not normally distributed, nor necessarily correlated in a linear fashion, we used the Spearman's rank correlation ρ. This statistic is the most commonly used non-parametric test for correlation [32]. All of the |ρ| were lower than 0.80: the covariates were not highly correlated and could be included simultaneously in a model [33]. Moreover, to determine whether the covariates should be considered as continuous or categorical, we examined the linearity of the association between each continuous covariate and the response variable (see Material S3). We finally obtained 4 continuous and 12 categorical candidate variables (Table 1). Only plausible two-way interactions were considered, *i.e.*, the interaction between temperature and rainfall at equivalent temporal lags, and the interaction between small ruminant density and dairy or beef cattle densities. Overall, 16 candidate covariates along with 4 plausible biological interactions between candidate variables were tested. The dataset was split randomly into “training” and “testing” subsets (75% and 25% of the data, respectively). We initially fitted a linear regression model to the training dataset using ordinary least squares (OLS). However, spatial autocorrelation in the residuals indicated that the assumption of independent errors was violated. We consequently extended the model to account for residual spatial structure by using a residuals autocovariate model (RAC) [34], which included, in addition to the environmental covariates, an autocovariate calculated from the residuals of the OLS fitted model (see Material S4). Statistical analyses were performed using the R v2.13.1 software [35]. Linear models were compared using the package MuMIn [36] and RAC models were fitted using the packages raster [37] and geoR [38].

We selected the best model by using backward model selection based on AICc [23]. As recommended by Burnham and Anderson [39], we considered that two nested models differing by less than 2 AICc points received identical support from the data. In such a situation, the model with fewer parameters was preferred. Once the best model was identified, we characterized its performance by using two statistics: the coefficient of determination and the Root Mean Squared Error (RMSE). The coefficient of determination, *i.e.*, the squared Pearson correlation *r* between predicted and observed values [40], [41] is a measure of the overall goodness of fit. We also calculated the Root Mean Squared Error (RMSE) [42] of values fitted to the training dataset since it is a good measure of prediction accuracy, lower values of RMSE indicating a better fit. Moreover, to evaluate the predictive power of the model we used the municipalities of the testing dataset. As for the training dataset, we calculated the squared Pearson correlation and the RMSE. Finally, we used likelihood ratio tests for nested models [43], [44] to assess the relative importance of environmental variables in the RAC model:
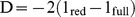
where D is the log-likelihood ratio test statistic, and l_red_ and l_full_ are the log-likelihoods of the reduced and full models, respectively. The full model is the RAC model and the reduced model contained all but one of the variables of the full model. The contribution of the omitted variable is thus evaluated, larger D values indicating a greater contribution to model fit.

## Results

**Figure 1:**
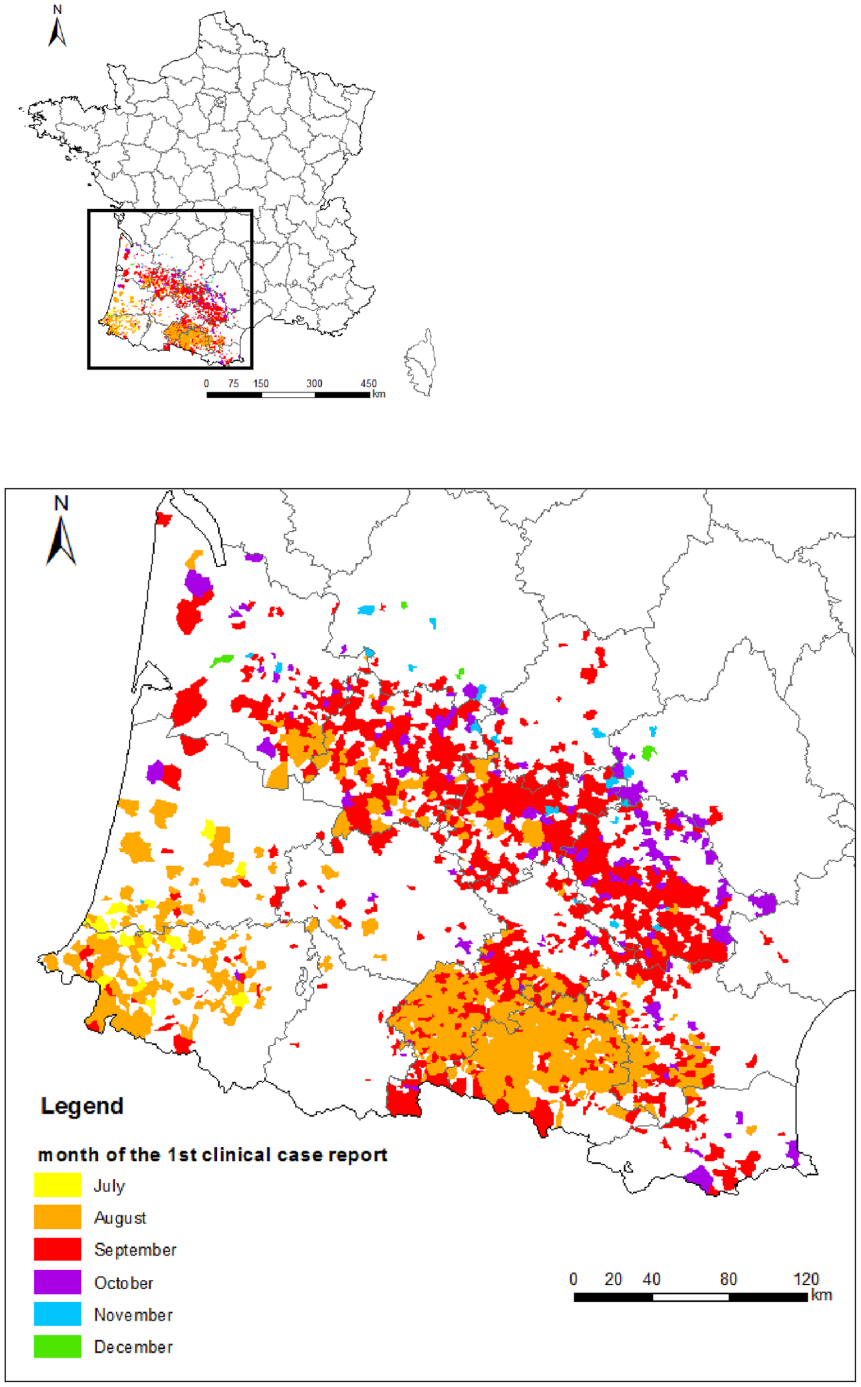
Dates of first BTV-1 clinical cases in 1,595 French municipalities, 2008. The colour corresponds to the month in which the first clinical case was reported in each municipality. One municipality had its first clinical case on 5^th^ January 2009 and is included in the December 2008 class.

Two BTV-1 clinical cases occurred in November 2007 in France. As in the BTV-8 study, we did not include these two first cases because they could induce bias in the estimation of the velocity of BTV-1 spread. These two cases occurred at the end of the vector activity period and were followed by the vector-free period during which BTV transmission was effectively inactive. In 2008, 4,195 BTV-1 clinical cases were reported. From this, we identified the date of the first clinical case in 1,595 municipalities (Fig. 1). These dates ranged from July 11^th^ 2008 to January 5^th^ 2009.

### 1 Velocity of BTV-1 spread estimation

**Figure 2:**
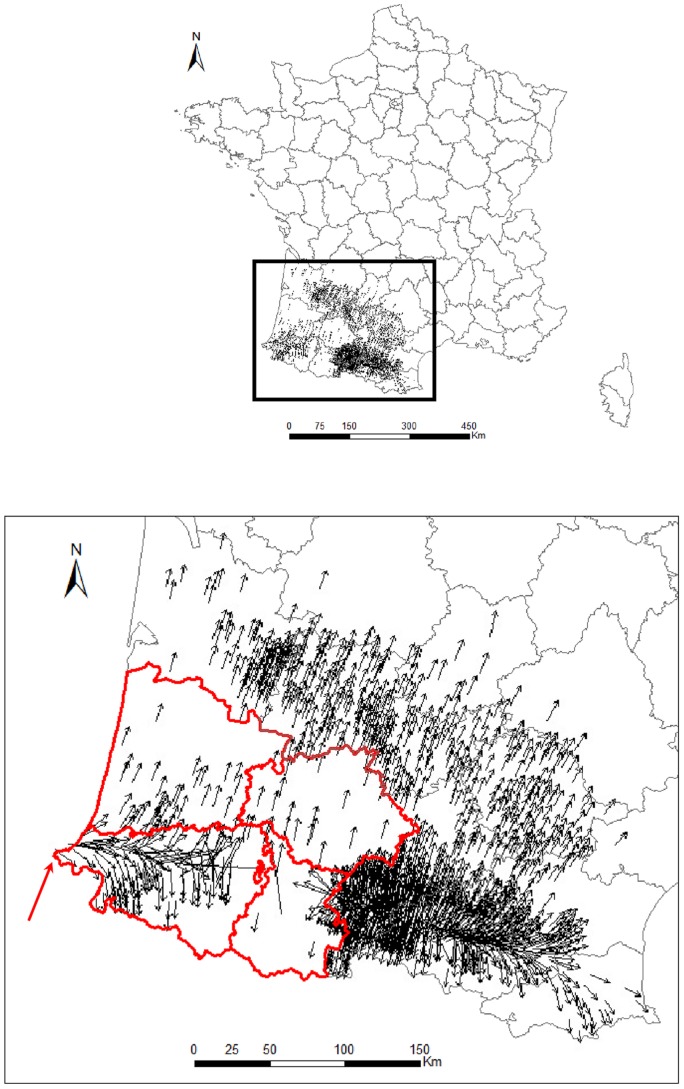
Velocity vectors for the 1,314 municipalities used to construct the RAC model. The length and direction of each arrow indicate the speed and direction of BTV-1 spread from each municipality centroid. The middle of the arrow is anchored at the municipality centroid. Administrative areas are mapped at the level of French departments. The initial area of BTV-1 introduction in France is indicated by a red arrow. The departments with an early vaccination scheme are displayed in red.

We selected the smallest subset of fourth-order TSA-SAR_err_ models for which the sum of the AICc weights was ≥0.9. The resulting subset contained 66 models (Table S2). Model-averaged parameters obtained from these 66 models were used to estimate a velocity for each of the 1,595 municipalities. These velocity estimates ranged from 0.98 to 126.34 km/day with a mean value of 5.35 km/day (median value  = 2.64 km/day). However, 90% (1,439) of the municipalities had velocity ≤10 km/day, indicating that BTV-1 spread was mostly local. High values for the velocity of BTV-1 spread were marginal, potentially linked with farm animal movements. Model residuals, *i.e.,* the difference between the fitted and observed date of first clinical case, had a mean non-significantly different from zero (0.2, 95% Confidence Interval (CI): −0.43−0.93) and a bell-shaped distribution. No spatial structure was detected in these residuals. The difference between the fitted and observed date of first clinical case was less than 16 days for 1,337 municipalities (84%) and environmental covariates were available for 1,314 of these municipalities (82%). For this sub-dataset of 1,314 municipalities the minimum and maximum velocities were identical to those of the full dataset, and the mean and median values of the velocity of BTV-1 spread were 5.72 and 2.74 km/day, respectively. The estimated velocities at these 1,314 municipalities were subsequently included in the analysis of the effect of vaccination (see section 3.2). Velocity vectors of the 1,314 municipalities are presented in Figure 2: from the initial introduction zone (indicated by a red arrow on the map), the virus spread rapidly from west to east along the Pyrenees Mountains, then, from this initial incursion, the virus spread sideways to the south and north. The departments with few infected municipalities, in red on the map, were departments with an early vaccination scheme.

### 2 Effect of vaccination on velocity of BTV-1 spread

**Figure 3:**
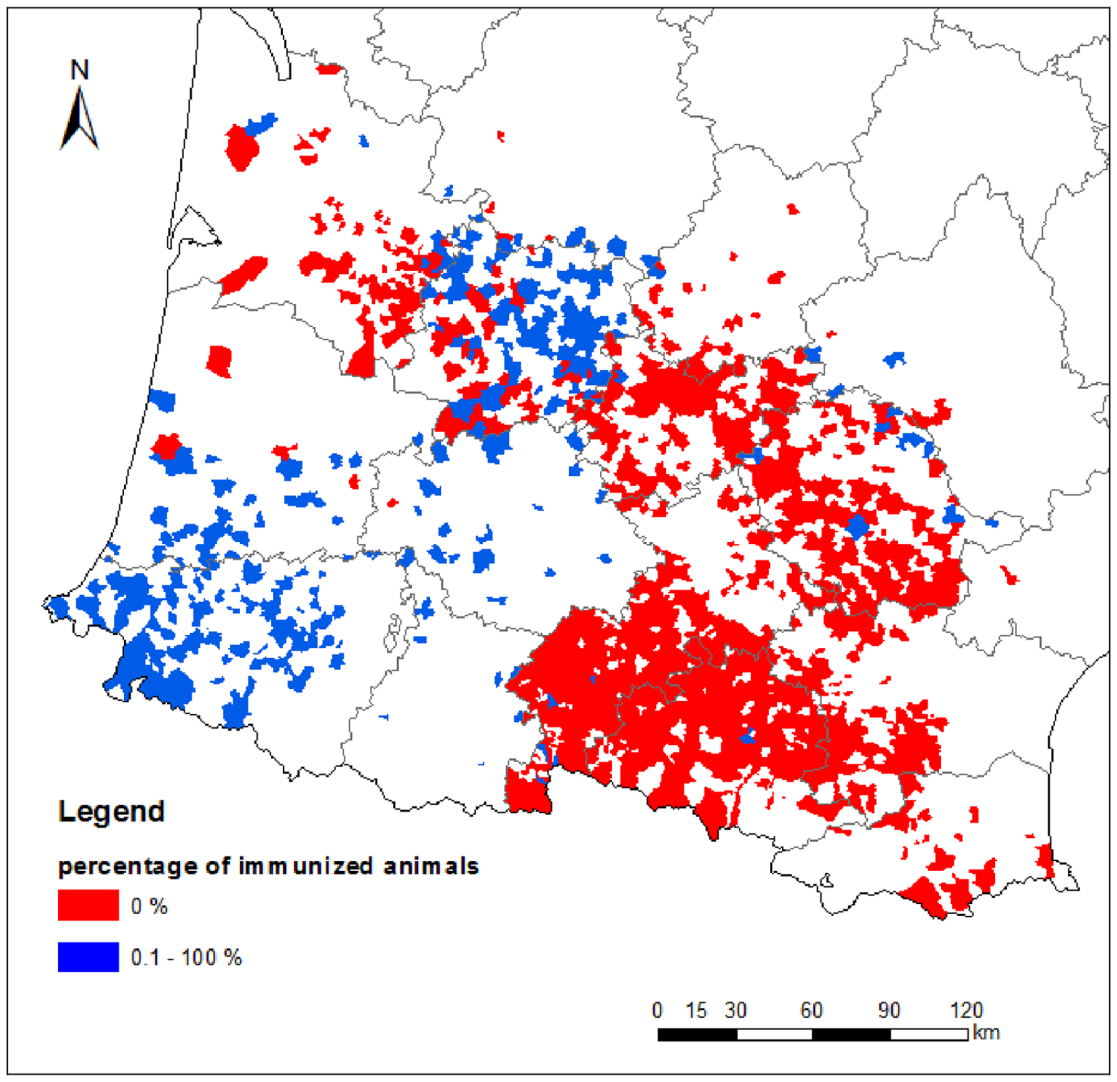
Vaccine coverage of the 1,314 municipalities used to study the velocity of BTV-1 spread. The percentage of immunized animals (cattle, sheep and goat), at the date of the first clinical case, is presented through colours.

Figure 3 displays vaccine coverage for the 1,314 municipalities used to analyse the effect of vaccination. Of these municipalities, 78% (1,028) had no vaccine coverage at the date of first clinical case. For the 286 municipalities with vaccine coverage, the percentage of immunized animals ranged from 0.4% to 100% (n = 15 municipalities) with a median value of 55%. The 1,314 municipality data subset was split randomly into “training” (986 municipalities) and “testing” subsets (328 municipalities). The first was used to fit linear regression models via OLS (Table S3). The best OLS model included elevation, edge density between arable land and forest, temperature at one and two month lags, rainfall at a two month lag, small ruminant and beef cattle densities, and vaccination coverage. This OLS model performed poorly in predicting the velocity of BTV-1 spread from environmental covariates (squared Pearson's *r* = 0.27, RMSE  = 7.33 km/day) in the training dataset and spatial autocorrelation at short distance was detected in the residuals (Fig. S2A). We thus fitted a RAC model to account for spatial autocorrelation. The RAC model contained the environmental covariates from the above OLS model plus an autocovariate that represented spatial autocorrelation in the residuals of the OLS model at a neighborhood size of 3.6 km. The fit of the RAC model was satisfactory (squared Pearson's r = 0.81, RMSE  = 3.69 km/day). In contrast to the OLS model, analysis of the residuals showed no spatial autocorrelation (Fig. S2). Parameter estimates of the RAC model are presented in Table 2. The RAC model was tested on the 328 municipality testing dataset and predictive performance was good (squared Pearson's r = 0.86, RMSE  = 3.14 km/day).

**Table 2:** Estimated coefficients, 95% confidence interval (CI) and p-values of the RAC model for the subset of 986 French municipalities.

covariates	class	coefficient	95%CI	p-value
intercept		3.45	1.990; 4.915	<0.001
elevation	b	1.10	0.396; 1.800	<0.01
	c	2.62	1.721; 3.513	<0.001
	d	1.87	0.820; 2.923	<0.001
DensBeef_Cattle	b	0.74	0.066; 1.406	<0.05
	c	1.33	0.647; 2.019	<0.001
	d	2.50	1.778; 3.223	<0.001
DensSmall_Ruminants	b	0.54	−0.139; 1.226	0.12
	c	−0.37	−1.075; 0.335	0.30
	d	−1.56	−2.296; −0.833	<0.001
VaccinCoverage	b	−1.66	−2.346; −0.975	<0.001
Tmax_lag1	b	1.99	1.263; 2.712	<0.001
	c	0.05	−0.742; 0.847	0.90
	d	−0.03	−0.852; 0.787	0.94
Rain_lag2	b	0.12	−0.561; 0.806	0.72
	c	1.69	0.972; 2.406	<0.001
	d	4.08	3.209; 4.951	<0.001
Tmax_lag2	b	−2.23	−2.993; −1.461	<0.001
	c	−4.40	−5.382; −3.421	<0.001
	d	−4.72	−5.829; −3.614	<0.001
arable-forest	b	2.70	2.013; 3.377	<0.001
	c	2.54	1.808; 3.268	<0.001
	d	1.61	0.856; 2.357	<0.001
autocovariate		1.09	1.049; 1.129	<0.001

See Table 1 for description of covariates. RAC model: Residuals Autocovariate model.

Estimated coefficients and p-values of environmental covariates are reported in Table 2. The intercept indicates an average velocity of BTV-1 spread of approximately 3.5 km/day (Table 2). As expected, vaccination was negatively associated with velocity of BTV-1 spread, which was, on average, 1.7 km/day lower in municipalities with immunized animals at the date of first clinical case, than in municipalities with no immunized animals.

Meteorological variables, landscape factors and host availability were also correlated to velocity of BTV-1 spread. The contribution of covariates to model fit was assessed via D-values of each covariate (Fig. S3). Weather at a two-month lag had the greatest effect on the velocity of BTV-1 spread, followed by edge density between arable land and forest, temperature at one-month lag and density of beef cattle. Weather at a two-month lag greatly influenced velocity, the latter being negatively correlated to the monthly average of maximum daily temperature such that a 4 km/day decrease in velocity was observed when monthly average of maximum daily temperature was higher than 25°C. Velocity was also positively associated with rainfall: heavy rainfall (>70 mm per month) increased the velocity by 4 km/day. Considering the effect of weather at a one-month lag, a monthly average of maximum daily temperature around 24°C was associated with a velocity increase of 1.9 km/day. Overall, the effect of weather on the velocity of BTV-1 spread was greater at a two-month lag than at a one-month lag. Regarding landscape-related variables, elevation and edge density between arable land and forest were positively correlated with velocity. Finally, velocity of BTV-1 spread was associated with beef cattle and small ruminant densities in different ways, while the density of dairy cattle had negligible effect. Velocity was positively associated with beef cattle density. On the other hand, the highest small ruminant densities (>20 small ruminants/km^2^) were negatively correlated with velocity.

**Figure 4:**
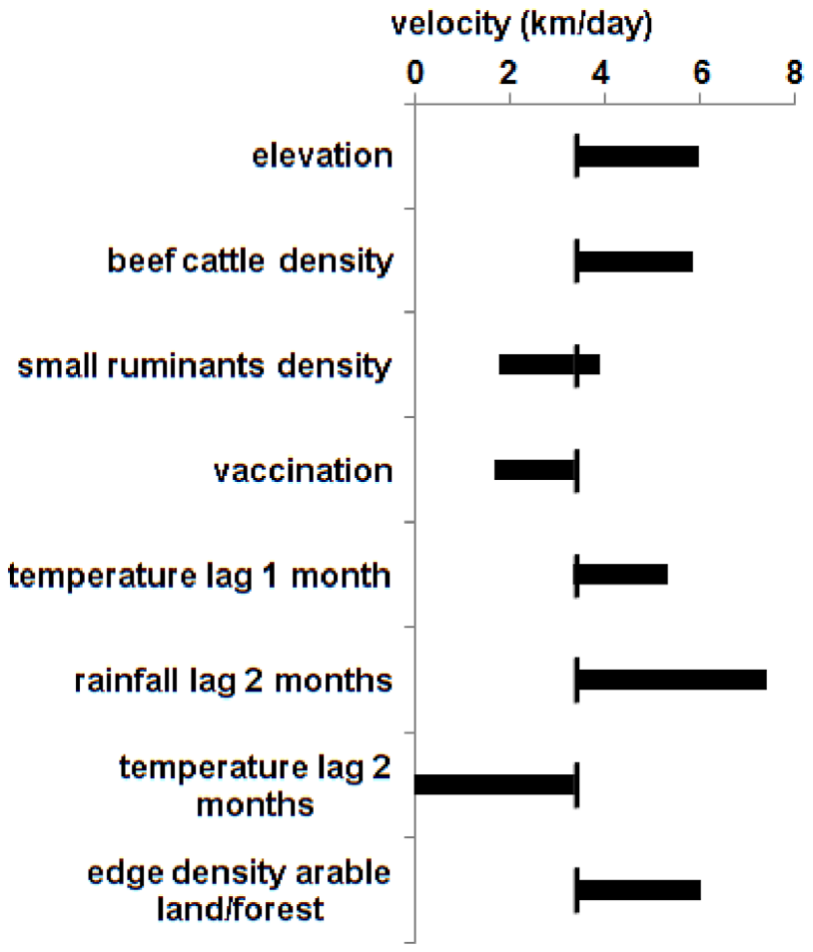
Estimated range of velocity variation as a function of covariates. Horizontal bars represent the range of velocities obtained when a single covariate is varied between its maximal and minimal observed value whilst all other covariates are held constant (see Table 2). The average velocity of 3.4 km/day is represented by the small vertical stroke crossing the horizontal bar. The velocities are estimated through the selected RAC model (n = 986 municipalities).

Finally, the range of velocities obtained by varying a single covariate across its observed range whilst holding all other covariates constant at their observed mean is presented in Figure 4. The graph provides a visual indication of the independent effect-size of each covariate on the average value of 3.5 km/day.

## Discussion

Several studies have modeled the effect of vaccination on BT spread and simulations have demonstrated that vaccination can be a highly effective means to control BT epizootics when a high level (>80%) of vaccine coverage is achieved [14], [15], [17], [18]. However, field studies investigating the effect of vaccination against BTV are rare. To our knowledge, our study is the first to have quantified vaccine induced reductions in the velocity of BTV spread using data from a real epidemic. After accounting for environmental factors known to influence the velocity of BT spread, vaccination divided by a factor 2 the mean velocity at which BTV-1 spread across the study area. Vaccination thus helped to slow down disease progression by decreasing the number of infectious hosts and vectors, and consequently the probability that infected vectors bite susceptible hosts in a non-contaminated area. Despite regulations on farm animal movements, BT has rapidly spread in Europe during the 2006–2008 BTV-1 and BTV-8 epizootics. Vaccination was the only efficient method that could stop BTV-8 and BTV-1 spread, and decrease the number of BT foci until apparent full eradication of BT among European livestock.

Since previous studies have estimated the velocity of BTV-8 spread in France and the effects of environmental covariates [23], [24], a comparison of BTV-1 and BTV-8 epizootics is possible. In both cases, restrictions were imposed on animal movements. Regarding the velocity of BT spread, the mean value of velocity of spread was similar for both serotypes (5.4 and 5.6 km/day for BTV-1 and BTV-8, respectively). The first and ninth deciles of the estimated velocities were 1.9 and 10.4 km/day for BTV-1 and 3.7 and 7.8 km/day for BTV-8, thus the distribution of estimated velocities appeared narrower for BTV-8 than for BTV-1. Moreover, the lower values of velocities that were observed for BTV-1 than for BTV-8 may be related to the effect of vaccination. Indeed, contrary to what was observed for BTV-1, there was no large area with high vaccine coverage for BTV-8. Regarding the influence of environmental factors, variables related to the ecology of *Culicoides* vectors (weather and elevation) were the main factors influencing the velocity of BT spread for both serotypes. Weather at a two month-lag plausibly could affect *Culicoides* abundance through direct effects on demographic life cycle parameters e.g. larvae and pupae require moist habitats, adults are prone to desiccation [3], and temperature is known to influence survival and duration of all stages of life cycle [45]. Weather at a one-month lag is most likely to influence *Culicoides* activity [46], [47]. The strong negative effect of temperature and the positive effect of rainfall, both at a two-month lag, suggest that in late summer (most clinical cases occurred in August and September) *Culicoides* dynamics in south-western France become damped when high temperatures exacerbate low level of moisture availability, a combination of factor which is known to induce low survival rates [48], [49]. However, at a one-month lag, monthly averages of maximum daily temperature around 24°C were associated with slightly increased velocities. These apparently contrasting results could reflect that incidence rates were greatest following several months of more or less exponential growth in both vector and virus populations and immediately prior to a desiccation induced crash in vector abundance effectively damping the velocity of further spread. Velocity of BTV-1 spread was also influenced by elevation, the highest velocity being observed for an elevation range between 280 and 454 m. The influence of elevation on velocity of BT spread, which was also observed for BTV-8 [23], was probably related to abundance, species composition and vector competence of the *Culicoides* vector populations. Indeed, *Culicoides* populations from the Obsoletus Complex have been found in Europe along a broad altitudinal cline [50], but their abundance changed with elevation. Moreover, Carpenter et al. [51] observed in the United Kingdom a variation of *Culicoides* susceptibility to BTV infection according to geographic areas within and between species and populations. Similar variation of *Culicoides* susceptibility and competence may partly explain the effect of elevation on velocity of BT spread. The positive effect of beef cattle density on BTV-1 spread contrasted with the negative effect of dairy cattle density on the BTV-8 spread. This might be related with differences in cattle management practices. Indeed, dairy cattle are kept close to farms, thus creating localized clusters of hosts and a relatively discontinuous pattern of host availability, which might be less favorable to BT spread. By contrast, beef cattle herds tend to be scattered throughout the landscape, a spatial arrangement that facilitates BTV progression [23]. High densities of small ruminants were negatively associated with the velocity of BTV-1 spread, a result that was also observed for BTV-8. With 1.3 million reproductive animals in 2008, dairy sheep farming was more important than meat sheep farming (926,000 reproductive animals) or goat farming (145,000 reproductive animals) in south-western France. Furthermore, according to our 2008 small ruminant count data, dairy sheep flocks are larger than meat sheep flocks, with a mean value of 153 *versus* 49 animals. Consequently, the negative association between high small ruminant densities and velocity may be due to dairy sheep management practices, which are similar to the dairy cattle management practices mentioned above. Another hypothesis would be that small ruminants were less competent hosts for BTV-1 than cattle, which may cause a dilution effect, and ultimately a negative association between high density of small ruminants and velocity of BTV-1 spread. One landscape-related covariate was significantly linked with velocity: the edge density between arable land and forests. This finding is consistent with previous results as edge density between arable land and forests was identified as a BTV-8 seropositivity risk factor for cattle in France [4]. It was also related to velocity of BTV-8 spread [23]. Arable land may serve as feeding areas for wildlife and forests provide breeding [52] and resting sites [50] for Obsoletus Complex midges. Edges between these habitats may facilitate contacts between BT vectors and wild hosts, then influencing BT dynamics.

Finally, two potential weaknesses of our study need to be considered. First, we used clinical cases to describe BT spread, and they may suffer from biases because of asymptomatic animals and, to a lesser degree, under-reporting of diseased animals. Consequently, the 1,595 municipalities included in the study might not represent an exhaustive sample of contaminated municipalities. Regarding asymptomatic animals, the severity of BT infection is influenced by various factors including host species, breed, age, individual susceptibility, environmental factors and BTV serotype [53]. Little information is available on BTV-1 clinical signs: the most common clinical signs observed in small ruminants are fever, depression, lethargy, facial edema and salivation [54]. However, a recent experimental study showed that BTV-1 infection induced more marked clinical signs in sheep than BTV-8 infection [55]. Moreover, as farmers received monetary compensation for BT diseased animals, under-reporting was probably rare. We could thus expect limited biases of BTV-1 clinical cases. Furthermore, even if the real BT clinical incidence was underestimated, it did not preclude an unbiased estimate of the spatial trend [56]. A second weakness of our study is that we did not account for wind-mediated vector movements on BT spread [57], [58]. However, our main purposes were to assess the effect of vaccination on BTV-1 spread velocity, and to compare the effect of environmental features on this velocity with previous results obtained for BTV-8. The effect of wind was beyond our scope.

## Conclusion

In this study we examined the effect of vaccination on the propagation velocity of BTV-1. For the first time, the effect of vaccination has been quantified using data obtained from a real BTV epizootic and after accounting for environmental factors known to modify the velocity of BT spread. Our findings emphasized the importance of vaccination in limiting disease spread across real agricultural landscapes. Finally, environmental factors should be accounted for when evaluating the role of vaccination on BT spread as they had a major influence.

## Supporting Information

Figure S1 **Timing of the vaccination campaign.** The period at which the first cattle (A) and small ruminants (B) were immunized is presented per department. The period is designed through the month of the year and a number that referred to the first half of the month (1) from the 1^st^ to the 15^th^, or the second half of the month (2) from the 16^th^ to the 31^st^, e.g. may_1 designed the period between the 1^st^ and the 15^th^ of May.
(DOC)

Figure S2 **A. Semi-variogram of the best OLS model (dashed line) and the RAC model (solid line).** B. Semi-variogram of the RAC model. The dashed lines represent the envelopes obtained from 999 permutations.
(PDF)

Figure S3 **Log-Likelihood ratio (D) statistics for environmental variables in the RAC model.** Larger D values indicate a greater contribution to model fit. P-values and parameter estimates of the RAC model are given in Table 2. See Table 1 for descriptions of environmental variables.
(PDF)

Table S1 **Spearman's rank correlations ρ among all the environmental covariates for the** **dataset with 1,314 municipalities.**
(PDF)

Table S2 **Degree of freedom (df), AICc, difference between AICc of each model and the** **minimum AICc score (ΔAICc) and Akaike weight (ω) of the 66 TSA-SAR_err_** **models used to obtain the model-averaged parameters for the 1,595 French** **municipalities.**
(PDF)

Table S3 **Degree of freedom (df), AICc, difference between AICc of each model and the minimum AICc score of the OLS models (ΔAICc) and Akaike weight (ω) of the 10 first OLS models and df and AICc of the RAC model fitted to explain the velocity of BTV-1 spread (n = 986 municipalities).**
(PDF)

Material S1 **Description of the method used to estimate the velocity of BT spread with a** **trend surface analysis model combined with a spatial error form of a** **simultaneous autoregressive model.**
(PDF)

Material S2 **Characteristics of the environmental covariates from the complete (1,595 municipalities) and restricted (1,314 municipalities) datasets.**
(PDF)

Material S3 **Description of the environmental covariates and their discretization.**
(PDF)

Material S4 **Description of the residuals autocovariate (RAC) model used to study the** **effect of environmental factors on the velocity of BT spread.**
(PDF)
