# Supplementary figures and images for: Did Vaccination Slow the Spread of Bluetongue in France?
Source: PLoS One. 2014 Jan 21;9(1):e85444. doi: 10.1371/journal.pone.0085444 (PMC3897431; doi:10.1371/journal.pone.0085444)

**Supplementary Figure S1.**

A


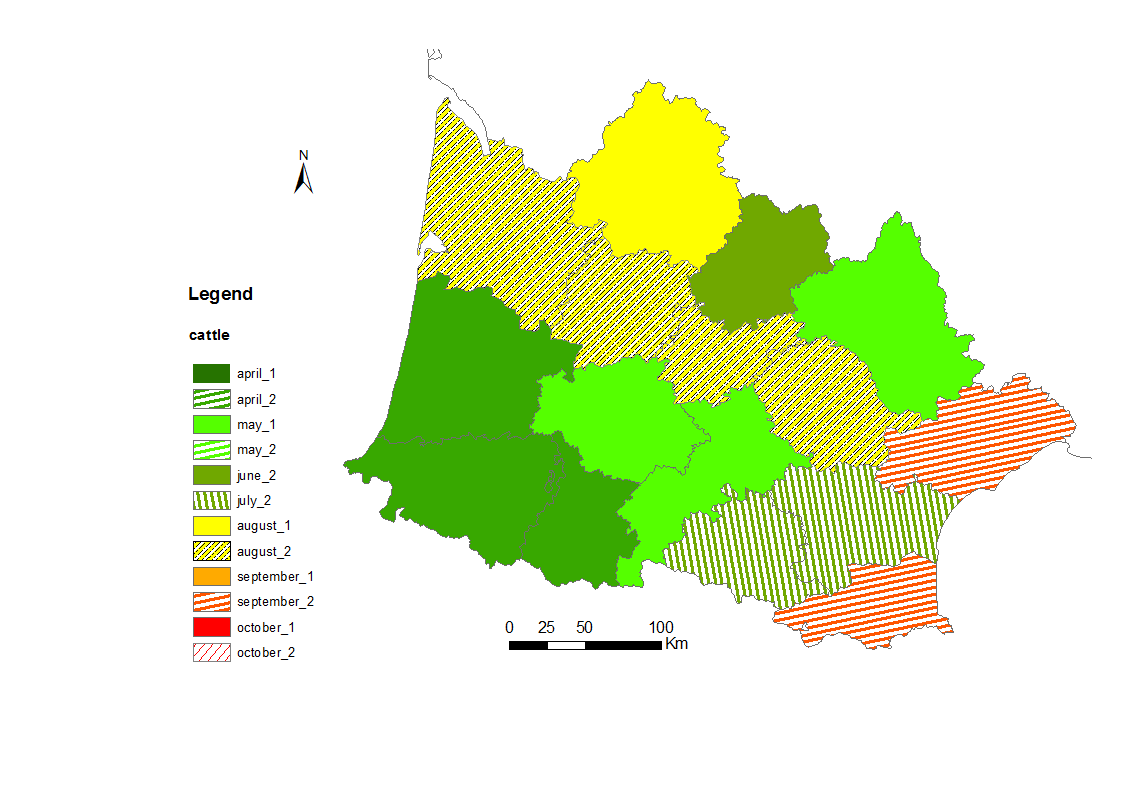


B


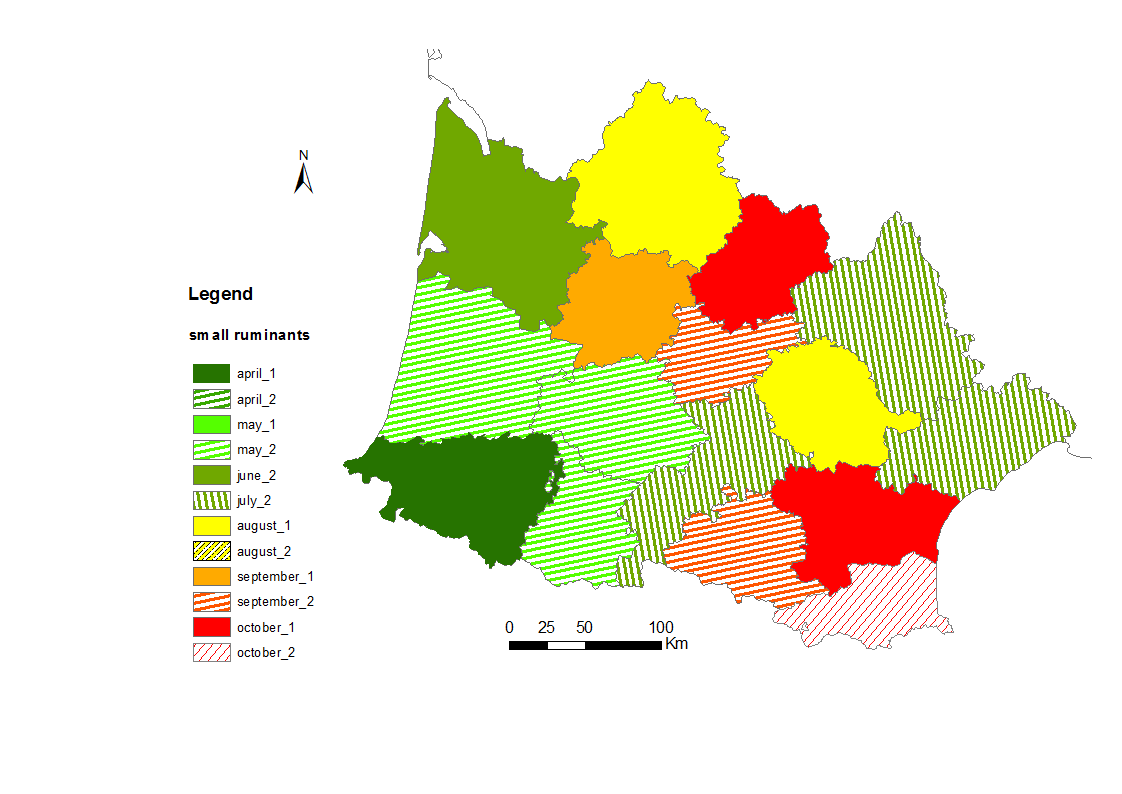

Supplement: Figure S1 — Timing of the vaccination campaign. The period at which the first cattle (A) and small ruminants (B) were immunized is presented per department. The period is designed through the month of the year and a number that referred to the first half of the month (1) from the 1st to the 15th, or the second half of the month (2) from the 16th to the 31st, e.g. may_1 designed the period between the 1st and the 15th of May. (DOC) [file pone.0085444.s001.doc]

**Supplementary Figure S2.**

**A**

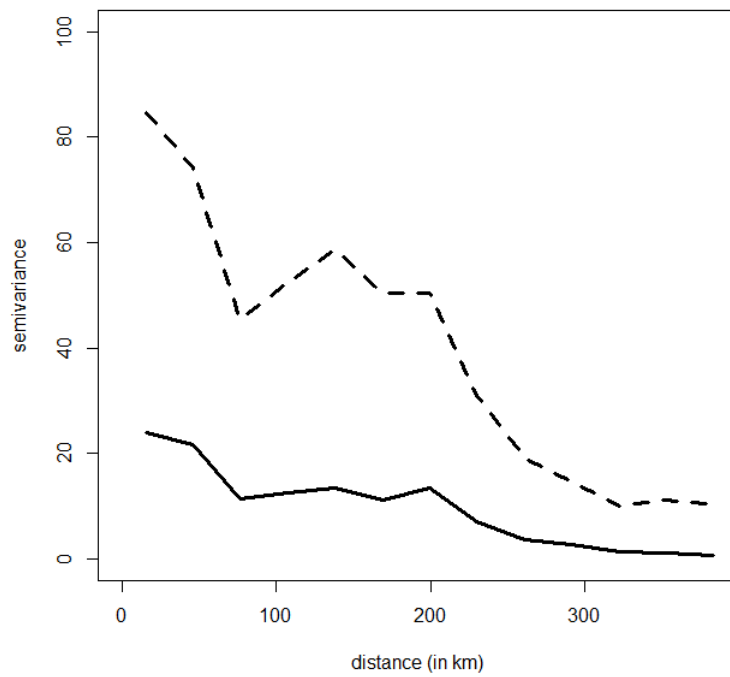

**B**

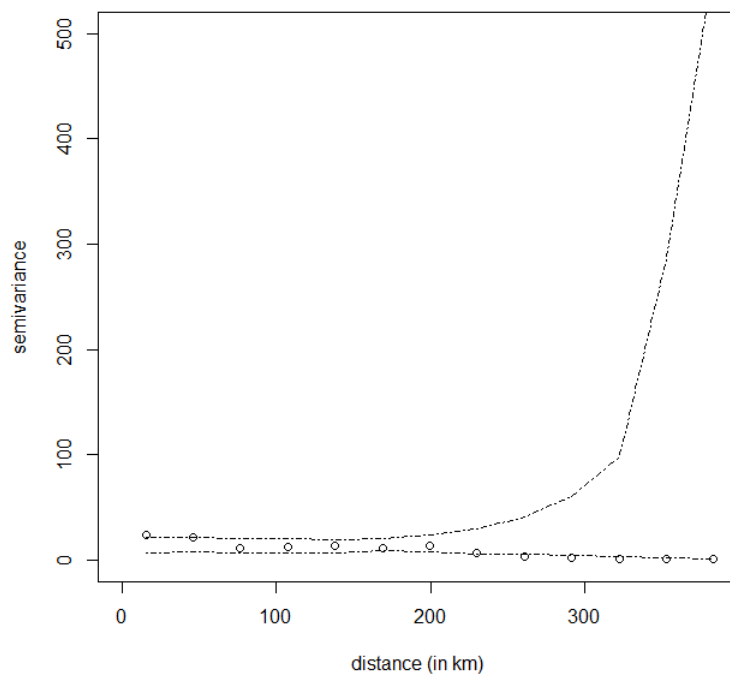

Supplement: Figure S2 — A. Semi-variogram of the best OLS model (dashed line) and the RAC model (solid line). B. Semi-variogram of the RAC model. The dashed lines represent the envelopes obtained from 999 permutations. (PDF) [file pone.0085444.s002.pdf]

**Supplementary Figure S3**

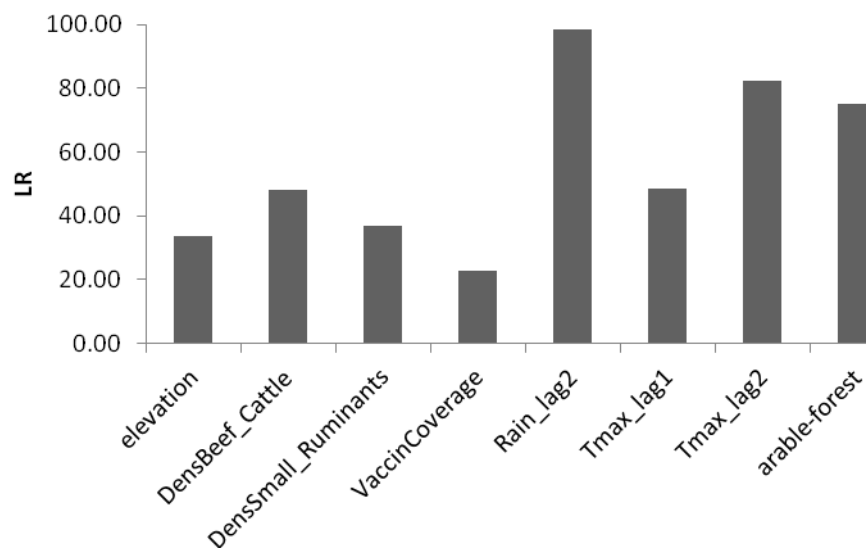

Supplement: Figure S3 — Log-Likelihood ratio (D) statistics for environmental variables in the RAC model. Larger D values indicate a greater contribution to model fit. P-values and parameter estimates of the RAC model are given in Table 2. See Table 1 for descriptions of environmental variables. (PDF) [file pone.0085444.s003.pdf]
