# Supplementary material for: Did Vaccination Slow the Spread of Bluetongue in France?
Source: PLoS One. 2014 Jan 21;9(1):e85444. doi: 10.1371/journal.pone.0085444 (PMC3897431; doi:10.1371/journal.pone.0085444)
Supplement: Table S1 — Spearman's rank correlations ρ among all the environmental covariates for the dataset with 1,314 municipalities. (PDF) [file pone.0085444.s004.pdf]

Supplementary Table S1. Spearman's rank correlations ρ among all the environmental covariates for the dataset with 1,314 municipalities. See Table 1 for descriptions of covariates. The two correlations ≥ 0.7 are highlighted in grey.

	elevation	Tmax_lag1	Tmax_lag2	Rain_lag1	Rain_lag2	DensBeef_Cattle	DensDairy_Cattle	DensSmall_Ruminants	SIDI	arable-forest	arable-pasture	forest-pasture	p_forest	p_arable	p_pasture
Tmax_lag1	-0.40														
Tmax_lag2	-0.59	0.55													
Rain_lag1	-0.27	-0.29	-0.12												
Rain_lag2	0.26	-0.29	-0.65	0.15											
DensBeef_Cattle	0.23	-0.15	-0.30	-0.06	0.22										
DensDairy_Cattle	-0.16	-0.01	0.03	0.04	0.00	0.38									
DensSmall_Ruminants	0.40	-0.18	-0.26	-0.16	0.16	0.23	0.06								
SIDI	-0.03	-0.08	-0.18	0.07	0.10	0.14	0.16	0.12							
arable-forest	-0.51	0.26	0.33	0.15	-0.20	-0.03	0.32	-0.24	0.16						
arable-pasture	-0.27	0.13	0.14	0.05	-0.12	0.20	0.35	-0.10	0.26	0.43					
forest-pasture	0.33	-0.21	-0.42	-0.01	0.30	0.33	0.12	0.30	0.38	-0.11	0.12				
p_forest	0.52	-0.36	-0.48	0.03	0.27	0.01	-0.21	0.25	0.14	-0.26	-0.36	0.50			

p_arable	-0.60	0.42	0.54	0.04	-0.29	-0.08	0.26	-0.37	-0.13	0.70	0.50	-0.40	-0.69		
p_pasture	0.23	-0.17	-0.33	-0.02	0.22	0.44	0.22	0.28	0.39	-0.13	0.42	0.78	0.13	-0.26	
VaccinCoverage	-0.37	-0.06	-0.10	0.40	0.15	0.10	0.22	-0.06	0.13	0.22	0.13	0.03	-0.10	0.13	0.09
