# Supplementary material for: Did Vaccination Slow the Spread of Bluetongue in France?
Source: PLoS One. 2014 Jan 21;9(1):e85444. doi: 10.1371/journal.pone.0085444 (PMC3897431; doi:10.1371/journal.pone.0085444)
Supplement: Table S2 — Degree of freedom (df), AICc, difference between AICc of each model and the minimum AICc score (ΔAICc) and Akaike weight (ω) of the 66 TSA-SARerr models used to obtain the model-averaged parameters for the 1,595 French municipalities. (PDF) [file pone.0085444.s005.pdf]

Supplementary Table S2. Degree of freedom (df), AICc, difference between AICc of each model and the minimum AICc score (ΔAICc) and Akaike weight (ω) of the 66 best TSA-SAR$_{err}$ models used to obtain the model-averaged parameters for the 1,595 French municipalities. The TSA-SAR$_{err}$ model is of the form:

$$t = \beta_0 + \beta_1 X + \beta_2 Y + \beta_3 X^2 + \beta_4 XY + \beta_5 Y^2 + \beta_6 X^3 + \beta_7 Y^3 + \beta_8 XY^2 + \beta_9 X^2Y + \beta_{10} X^4 + \beta_{11} Y^4 + \beta_{12} XY^3 + \beta_{13} X^3Y + \beta_{14} X^2Y^2 + \lambda W\mu + \varepsilon.$$

t is the number of days to the BTV-1 introduction in France on November 10th 2007, β_i are the fitted parameters, X and Y are the geographic coordinates of the municipality centroids centralised around the putative municipality of BTV-1 introduction in France, the term ($\lambda W\mu$) represents the spatial structure (λW) in the spatially dependent error term (μ) and ε represents the error term. Parameters included and excluded within each model are indicated by grey and white cells respectively.

Model #	X	Y	X^2	Y^2	X^3	Y^3	X^4	Y^4	XY	XY^2	X^2Y	XY^3	X^3Y	X^2Y^2	df	AICc	ΔAICc	ω
1	■	■	■	■	■	■		■	■	■	■	■	■		15	12937	0.00	0.093
2	■	■	■	■	■	■		■	■	■		■	■	■	15	12938	0.37	0.077
3	■	■	■	■	■	■	■	■	■	■	■	■	■		16	12938	1.13	0.053
4	■	■	■	■	■	■		■	■		■		■	■	14	12938	1.21	0.051
5	■	■	■	■	■	■	■	■	■	■		■	■	■	16	12939	1.79	0.038
6	■	■	■	■	■	■		■	■	■	■	■	■	■	16	12939	2.02	0.034
7	■	■	■	■	■	■	■	■	■		■		■	■	15	12940	2.29	0.030
8	■	■	■	■	■	■		■	■		■	■	■	■	15	12940	2.50	0.027
9	■	■	■	■	■	■		■	■	■	■		■	■	15	12940	2.74	0.024

10														14	12940	2.79	0.023
11														14	12940	2.89	0.022
12														17	12940	3.01	0.021
13														16	12940	3.02	0.020
14														14	12940	3.07	0.020
15														13	12940	3.22	0.019
16														14	12941	3.32	0.018
17														16	12941	3.33	0.018
18														14	12941	3.72	0.014
19														14	12941	3.84	0.014
20														14	12941	3.85	0.014
21														14	12941	3.97	0.013
22														15	12942	4.36	0.010
23														13	12942	4.41	0.010
24														14	12942	4.46	0.010
25														15	12942	4.52	0.010
26														13	12942	4.68	0.009
27														13	12942	4.75	0.009
28														15	12942	4.82	0.008
29														15	12942	4.82	0.008
30														15	12942	4.90	0.008
31														13	12942	5.02	0.008
32														13	12942	5.02	0.008
33														13	12942	5.03	0.008
34														13	12942	5.07	0.007
35														14	12942	5.14	0.007

36															13	12942	5.15	0.007
37															13	12942	5.18	0.007
38															13	12942	5.19	0.007
39															13	12942	5.21	0.007
40															13	12943	5.31	0.007
41															13	12943	5.38	0.006
42															13	12943	5.39	0.006
43															13	12943	5.44	0.006
44															13	12943	5.51	0.006
45															14	12943	5.55	0.006
46															15	12943	5.56	0.006
47															12	12943	5.64	0.006
48															15	12943	5.67	0.005
49															12	12943	6.04	0.005
50															13	12943	6.08	0.004
51															13	12943	6.18	0.004
52															13	12943	6.19	0.004
53															15	12943	6.20	0.004
54															14	12944	6.26	0.004
55															14	12944	6.60	0.003
56															16	12944	6.77	0.003
57															13	12944	6.81	0.003
58															14	12944	6.85	0.003
59															15	12944	6.90	0.003
60															13	12944	6.92	0.003
61															14	12944	6.93	0.003

62															14	12944	6.96	0.003
63															14	12944	6.98	0.003
64															14	12944	7.04	0.003
65															14	12944	7.09	0.003
66															12	12944	7.10	0.003
