# Supplementary material for: Did Vaccination Slow the Spread of Bluetongue in France?
Source: PLoS One. 2014 Jan 21;9(1):e85444. doi: 10.1371/journal.pone.0085444 (PMC3897431; doi:10.1371/journal.pone.0085444)
Supplement: Table S3 — Degree of freedom (df), AICc, difference between AICc of each model and the minimum AICc score of the OLS models (ΔAICc) and Akaike weight (ω) of the 10 first OLS models and df and AICc of the RAC model fitted to explain the velocity of BTV-1 spread (n = 986 municipalities). (PDF) [file pone.0085444.s006.pdf]

Supplementary Table S3. Degree of freedom (df), AICc, difference between AICc of each model and the minimum AICc score of the OLS models (ΔAICc) and Akaike weight (ω) of the 8 OLS models fitted from the 986 municipalities to explain the velocity of BTV-1 spread with greatest weight of evidence. The selected OLS model is highlighted in grey. The RAC model was obtained from the selected OLS model after adding an autocovariate term (see Materials and Methods and Supplementary Material S3 for details). See Table 1 for descriptions of covariates.

covariates	OLS #1	OLS #2	OLS #3	OLS #4	OLS #5	OLS #6	OLS #7	OLS #8	**RAC**
elevation	+	+	+	+	+	+	+	+	+
Rain_lag1	+	+	+	+		+	+	+	
Rain_lag2	+	+	+	+	+	+	+	+	+
Tmax_lag1	+	+	+	+	+	+	+	+	+
Tmax_lag2	+	+	+	+	+	+	+	+	+
DensBeef_Cattle	+	+	+	+	+	+	+	+	+
DensDairy_Cattle									
DensSmall_Ruminants	+	+	+	+	+	+	+	+	+
VaccinCoverage	+	+	+	+	+	+	+	+	+
arable-pasture									
arable-forest	+	+	+	+	+	+	+	+	+
forest-pasture									

SIDI			+			+			
p_arable			+					+	
p_pasture									
p_forest			+	+			+		
Rain_lag1 × Tmax_lag1	+	+	+	+		+	+	+	
Rain_lag2 × Tmax_lag2		+					+		
DensBeef_Cattle × DensSmall_Ruminants									
DensDairy_Cattle × DensSmall_Ruminants									
autocovariate									+
df	36	44	39	37	24	37	45	37	**26**
AICc	6773.7	6774.4	6774.9	6774.9	6775.1	6775.1	6775.6	6775.6	**5424.0**
ΔAICc	0.00	0.66	1.13	1.19	1.33	1.40	1.84	1.84	*
ω	0.08	0.06	0.05	0.05	0.04	0.04	0.03	0.03	*

*ΔAICc and ω are not accurate for the RAC model as they were calculated for the set of OLS models. AICc and df of the RAC model were presented here for comparison with OLS models.
