# Supplementary material for: Did Vaccination Slow the Spread of Bluetongue in France?
Source: PLoS One. 2014 Jan 21;9(1):e85444. doi: 10.1371/journal.pone.0085444 (PMC3897431; doi:10.1371/journal.pone.0085444)
Supplement: Material S1 — Description of the method used to estimate the velocity of BT spread with a trend surface analysis model combined with a spatial error form of a simultaneous autoregressive model. (PDF) [file pone.0085444.s007.pdf]

**Supplementary Material S1: Description of the method used to estimate the velocity of BTV-1 spread with a trend surface analysis model combined with a spatial error form of a simultaneous autoregressive model.**

To estimate the velocity of BTV-1 spread we combined a fourth order Trend Surface Analysis (TSA) model with a spatial error form of a Simultaneous Autoregressive ($SAR_{err}$) model. This combination leads to a model that takes the form

$$t = \beta_0 + \beta_1 X + \beta_2 Y + \beta_3 X^2 + \beta_4 XY + \beta_5 Y^2 + \beta_6 X^3 + \beta_7 X^2 Y + \beta_8 XY^2 + \beta_9 Y^3 + + \beta_{10} X^4 + \beta_{11} X^3 Y + \beta_{12} XY^3 + \beta_{13} X^2 Y^2 + \beta_{14} Y^4 + \lambda W\mu + \varepsilon.$$

In this model t is the number of days to the first French BTV-1 case, which was reported on 10 November 2007, $\beta_i$ are the fitted parameters, X and Y the geographic coordinates of the municipality adjusted to the localization of the first French BTV-1 case on 10 November 2007, $\lambda$ is the spatial autoregression coefficient, W is the spatial weights matrix, $\mu$ represents the spatially dependent error term, and $\varepsilon$ represents the spatially independent error term.

The use of the TSA-$SAR_{err}$ model allowed accounting for the spatial autocorrelation in residuals found at an 80-km distance. We thus used a neighbourhood matrix with a variance stabilizing ‘S’ coding style [1], a neighbourhood distance of 80 km, and the weights set to be proportional to the inverse distance between the centroids of the municipalities. As recommended by Lichstein et al. [2], we chose as neighbourhood distance the distance until which the residuals from the TSA were autocorrelated, *i.e.*, 80 km.

We used a model averaging procedure to account for model selection uncertainty and obtain robust estimates of model parameters [3]. We based the model selection on the Akaike Information Criterion corrected for small sample size (AICc) to select the best model based on both model fit and model complexity [4]. We applied the function “dredge” from the “MuMIn” R package to the full fourth order TSA-$SAR_{err}$ model to obtain the AICc table of all

the nested models. We also got the Akaike weights $\omega_i$, which provides a relative weight of evidence for each model [3]. A weighted average of parameter estimates $\beta_i$ is obtained by averaging $\hat{\beta}_i$ across all models:

$$\tilde{\tilde{\beta}}_i = \sum_{j=1}^{R} \omega_j \hat{\beta}_{ij}$$

where $\beta_i$ = 0 if the variable is not included in model $j$. From the AICc table we selected the models to sum the Akaike weights from largest to smallest until that sum was ≥0.9. From this subset of models we obtained the model-averaged parameters (function avg.model of the MuMIn package [5]). A model-averaged estimator has better precision and reduced bias compared with the estimator of that parameter from just the selected best model [6]. The BT front-wave velocities were calculated using the model-averaged parameters (see Pioz et al. 2011 [7] for details and references related to the method). Velocity vector arrows were mapped to depict the instantaneous velocity at each municipality centroids. The vector field provides a visual representation of the pattern of direction and velocities of BT spread over the entire region [8]. The plotted velocity vectors are the square root of the values of velocity for easier visualization. Longer is the arrow, higher is the velocity.

All of the statistical analyses were realized using R software v2.13.1 [9]: model averaging were realized with the package MuMIn [5] and spatial models and variograms were fitted using the packages spdep [10] and geoR [11], respectively. The velocity vectors were plotted using the function vectorField of the R package plotrix [12].


1. Bivand RS, Pebesma EJ, Gómez-Rubio V (2008) Applied spatial data analysis with R.; Gentleman R, Hornik K, Parmigiani G, editors. New-York: Springer. 378 p.
2. Lichstein JW, Simons TR, Shriner SA, Franzreb KE (2002) Spatial autocorrelation and autoregressive models in ecology. Ecological Monographs 72: 445-463.
3. Burnham KP, Anderson DR (2004) Multimodel inference: understanding AIC and BIC in model selection. Sociological Methods and Research 33: 261-304.

4. Burnham KP, Anderson DR (2002) Model selection and Multi-Model Inference, a practical information-theoretic approach. New-York: Springer-Verlag. 488 p.
5. R package version 1.7.7 (2012) MuMIn: Multi-model inference
6. Burnham KP, Anderson DR (2001) Kullback-Leibler information as a basis for a strong inference in ecological studies. Wildlife Research 28: 111-119.
7. Pioz M, Guis H, Calavas D, Durand B, Abrial D, et al. (2011) Estimating front-wave velocity of infectious diseases: a simple, efficient method applied to bluetongue. Veterinary Research 42: 60.
8. Lucey BT, Russell CA, Smith D, Wilson ML, Long A, et al. (2002) Spatiotemporal analysis of epizootic raccoon rabies propagation in Connecticut, 1991-1995. Vector borne and zoonotic diseases 2: 77-86.
9. R Development Core Team (2010) The R Foundation for Statistical Computing: R statistical software. Vienna, Austria
10. R package version 0.5-37 (2011) Package spdep: spatial dependence: weighting schemes, statistics, and models
11. R package version 1.6-35 (2001) geoR: a package for geostatistical analysis
12. R package version 3.4-5 (2012) plotrix: Various plotting functions
