# Supplementary material for: Did Vaccination Slow the Spread of Bluetongue in France?
Source: PLoS One. 2014 Jan 21;9(1):e85444. doi: 10.1371/journal.pone.0085444 (PMC3897431; doi:10.1371/journal.pone.0085444)
Supplement: Material S2 — Characteristics of the environmental covariates from the complete (1,595 municipalities) and restricted (1,314 municipalities) datasets. (PDF) [file pone.0085444.s008.pdf]

characteristics of the covariates
in the dataset with 1,595 municipalities

xxDis
Min. :244380
1st Qu.:419030
Median :466380
Mean :461013
3rd Qu.:522630
Max. :628480

yyDis
Min. :1524070
1st Qu.:1605770
Median :1643070
Mean :1661315
3rd Qu.:1718770
Max. :1872270

1st_clinical_case
Min. :153.0
1st Qu.:198.0
Median :210.0
Mean :211.7
3rd Qu.:223.0
Max. :331.0

week
Min. :28.00
1st Qu.:35.00
Median :36.00
Mean :36.66
3rd Qu.:38.00
Max. :54.00

elevation
Min. : 4.0
1st Qu.: 130.0
Median : 261.0
Mean : 345.5
3rd Qu.: 433.5
Max. :2044.0

c_elevation
a:386
b:288
c:372
d:549

Tmax_lag1

characteristics of the covariates
in the dataset with 1,314 municipalities

xxDis
Min. :244380
1st Qu.:423205
Median :467530
Mean :462578
3rd Qu.:521955
Max. :628480

yyDis
Min. :1524070
1st Qu.:1603870
Median :1636270
Mean :1656667
3rd Qu.:1710595
Max. :1852470

1st_clinical_case
Min. :173.0
1st Qu.:197.0
Median :207.0
Mean :208.9
3rd Qu.:220.0
Max. :258.0

week
Min. :31.00
1st Qu.:35.00
Median :36.00
Mean :36.25
3rd Qu.:38.00
Max. :43.00

elevation
Min. : 4.0
1st Qu.: 144.2
Median : 279.0
Mean : 358.9
3rd Qu.: 453.0
Max. :2044.0

c_elevation
a:329
b:331
c:327
d:327

Tmax_lag1

Min.   : 5.52
1st Qu.:23.40
Median :24.26
Mean   :23.86
3rd Qu.:25.10
Max.   :28.71

Tmax_lag2
Min.   :11.54
1st Qu.:23.14
Median :24.67
Mean   :24.32
3rd Qu.:25.76
Max.   :29.80

Rain_lag1
Min.   : 5.40
1st Qu.: 35.00
Median : 46.00
Mean   : 48.36
3rd Qu.: 59.95
Max.   :154.60

Rain_lag2
Min.   : 6.20
1st Qu.: 37.05
Median : 48.30
Mean   : 56.98
3rd Qu.: 68.50
Max.   :295.90

c_Tmax_lag1
a:399
b:399
c:399
d:398

c_Tmax_lag2
a:399
b:399
c:399
d:398

c_Rain_lag1
a:399
b:399
c:399
d:398

c_Rain_lag2

Min.   :13.90
1st Qu.:23.70
Median :24.40
Mean   :24.12
3rd Qu.:25.20
Max.   :28.70

Tmax_lag2
Min.   :15.20
1st Qu.:23.10
Median :24.70
Mean   :24.39
3rd Qu.:25.80
Max.   :29.00

Rain_lag1
Min.   : 7.60
1st Qu.: 35.30
Median : 46.10
Mean   : 47.72
3rd Qu.: 59.30
Max.   :120.30

Rain_lag2
Min.   : 6.20
1st Qu.: 37.23
Median : 48.65
Mean   : 56.77
3rd Qu.: 69.30
Max.   :206.70

c_Tmax_lag1
a:323
b:331
c:330
d:330

c_Tmax_lag2
a:336
b:346
c:316
d:316

c_Rain_lag1
a:320
b:331
c:328
d:335

c_Rain_lag2

a:399
b:399
c:399
d:398

DensBeefCattle
Min.   : 0.000
1st Qu.: 2.942
Median : 11.870
Mean   : 20.136
3rd Qu.: 27.413
Max.   :207.406

DensDairyCattle
Min.   : 0.000
1st Qu.: 0.000
Median : 1.162
Mean   : 5.769
3rd Qu.: 7.734
Max.   :134.994

DensSmallRum
Min.   : 0.0000
1st Qu.: 0.9265
Median : 4.7190
Mean   : 25.4646
3rd Qu.: 20.0415
Max.   :915.5640

c_DensBeefCattle
a:403
b:400
c:389
d:403

c_DensDairyCattle
a:410
b:367
c:427
d:391

c_DensSmallRum
a:419
b:331
c:445
d:400

SIDI
Min.   :0.0000
1st Qu.:0.5100

a:320
b:330
c:336
d:328

DensBeefCattle
Min.   : 0.00
1st Qu.: 3.80
Median : 12.85
Mean   : 21.30
3rd Qu.: 28.90
Max.   :207.40

DensDairyCattle
Min.   : 0.000
1st Qu.: 0.000
Median : 1.300
Mean   : 5.938
3rd Qu.: 8.100
Max.   :135.000

DensSmallRum
Min.   : 0.00
1st Qu.: 1.10
Median : 5.35
Mean   : 26.51
3rd Qu.: 22.23
Max.   :915.60

c_DensBeefCattle
a:336
b:325
c:325
d:328

c_DensDairyCattle
a:319
b:336
c:327
d:332

c_DensSmallRum
a:305
b:330
c:335
d:344

SIDI
Min.   :0.0000
1st Qu.:0.5100

```
Median :0.6165
Mean   :0.5832
3rd Qu.:0.6893
Max.   :0.8403

  arable-forest
Min.   : 0.0000
1st Qu.: 0.0000
Median : 0.2774
Mean   : 1.4762
3rd Qu.: 1.9806
Max.   :18.7230

  arable-pasture
Min.   : 0.0000
1st Qu.: 0.0000
Median : 0.0000
Mean   : 0.6407
3rd Qu.: 0.6212
Max.   :15.0043

  forest-pasture
Min.   : 0.0000
1st Qu.: 0.0000
Median : 0.7165
Mean   : 2.2628
3rd Qu.: 3.4110
Max.   :32.1656

    p_arable
Min.   :  0.00
1st Qu.:  0.00
Median : 12.25
Mean   : 24.65
3rd Qu.: 45.32
Max.   :100.00

   p_pasture
Min.   : 0.00000
1st Qu.: 0.01705
Median : 6.81445
Mean   :11.24721
3rd Qu.:17.71449
Max.   :82.69791

   p_forest
Min.   : 0.000
1st Qu.: 8.068
Median :20.576
Mean   :25.918
```

```
Median :0.6155
Mean   :0.5827
3rd Qu.:0.6885
Max.   :0.8403

  arable-forest
Min.   : 0.000
1st Qu.: 0.000
Median : 0.270
Mean   : 1.509
3rd Qu.: 2.078
Max.   :18.720

  arable-pasture
Min.   : 0.0000
1st Qu.: 0.0000
Median : 0.0000
Mean   : 0.6608
3rd Qu.: 0.6650
Max.   :15.0000

  forest-pasture
Min.   : 0.000
1st Qu.: 0.000
Median : 0.785
Mean   : 2.315
3rd Qu.: 3.525
Max.   :32.170

    p_arable
Min.   :  0.00
1st Qu.:  0.00
Median : 12.12
Mean   : 24.70
3rd Qu.: 45.55
Max.   :100.00

   p_pasture
Min.   : 0.0000
1st Qu.: 0.1496
Median : 7.1940
Mean   :11.5910
3rd Qu.:18.0731
Max.   :82.6979

   p_forest
Min.   : 0.000
1st Qu.: 8.235
Median :20.425
Mean   :26.173
```

3rd Qu.:39.398	3rd Qu.:40.174
Max. :89.442	Max. :89.442
c_arable-forest	c_arable-forest
a:685	a:573
b:303	b:256
c:303	c:243
d:304	d:242
c_arable-pasture	c_arable-pasture
a:925	a:750
b:223	b:185
c:223	c:188
d:224	d:191
c_forest-pasture	c_forest-pasture
a:560	a:445
b:345	b:288
c:345	c:289
d:345	d:292
VaccinCoverage	VaccinCoverage
Min. : 0.00	Min. : 0.00
1st Qu.: 0.00	1st Qu.: 0.00
Median : 0.00	Median : 0.00
Mean : 13.49	Mean : 11.78
3rd Qu.: 0.00	3rd Qu.: 0.00
Max. :100.00	Max. :100.00
NA's :31	
c_VaccinCoverage	c_VaccinCoverage
a:1174	a:1028
b: 390	b: 286
NA's: 31	
Predicted_1stClinical_case	Predicted_1stClinical_case
Min. :182.0	Min. :182.0
1st Qu.:201.3	1st Qu.:201.1
Median :209.1	Median :207.9
Mean :211.5	Mean :210.8
3rd Qu.:221.6	3rd Qu.:220.7
Max. :247.5	Max. :246.8
velocity	velocity
Min. : 0.9847	Min. : 0.9847
1st Qu.: 2.2240	1st Qu.: 2.2310
Median : 2.6399	Median : 2.7403
Mean : 5.3539	Mean : 5.7213
3rd Qu.: 4.9335	3rd Qu.: 5.3670
Max. :126.3382	Max. :126.3382
