# Supplementary material for: Did Vaccination Slow the Spread of Bluetongue in France?
Source: PLoS One. 2014 Jan 21;9(1):e85444. doi: 10.1371/journal.pone.0085444 (PMC3897431; doi:10.1371/journal.pone.0085444)
Supplement: Material S3 — Description of the environmental covariates and their discretization. (PDF) [file pone.0085444.s009.pdf]

Supplementary Material S3: description of the environmental covariates and their discretization

Host availability variables. Density of beef cattle and dairy cattle in September 2008 was obtained from the French National Cattle Register (BDNI). Because of maternal immunity, we only considered cattle over 2 months old to reflect the population size of susceptible hosts. Small ruminant density in January 2008 was obtained from the non exhaustive census realized every year in January by the French Ministry of Agriculture. Densities were expressed in number of animals per km^2. A change in the host density is expected to modify the velocity of BT spread as it will change the probability of transmission from vector to host. Furthermore, different host species may change the velocity in different ways because of differences in susceptibility, capability to transmit the disease and management practices. Consequently, the density of small ruminant, beef cattle and dairy cattle were not expected to have the same influence on the velocity of BT spread.

Elevation. Average elevation of each municipality measured in meters above sea level was extracted from the database GEOFLA® 2002, edition 6, of the Institut Géographique National (IGN). Average elevation is calculated by IGN from elevation obtained at a resolution of 50 meters. Elevation may influence the velocity of BT spread through its effect on vector abundance and *Culicoides* species composition.

Landscape-related variables.

Municipality-specific land cover data were extracted from the 2006 version of the CORINE (Coordination de l’Information sur l’Environnement) Land Cover database (CLC), provided by the European Environment Agency at a resolution of 100 meters [41]. Landscape

components may be important in defining favourable environments for BT [28]. Durand et al. [21] identified three landscape associated with BTV-8 seropositivity: forests, pastures and arable land. Furthermore, the breeding habitat of *Culicoides obsoletus* sensu stricto (and possibly *C. scoticus*), the major vector involved in BTV transmission in northern Europe, is the leaf litter of deciduous trees such as the common beech *Fagus sylvatica* [42,43]. We consequently discarded coniferous forests from the analysis and extracted from the 44 CLC landscape classes the proportion of the municipality area covered by deciduous and mixed forests, arable land and pastures. For each pair of these classes, we calculated the edge density, which is the length of the edges between two classes divided by the municipality area (3 variables in m/hectare). Finally, we calculated an index of diversity to characterize the landscape diversity in each municipality. Simpson's Diversity Index (SIDI) is a popular diversity measure in community ecology [44]. It has an intuitive interpretation as it represents the probability that any 2 randomly selected pixels would be different patch types. SIDI = 0 when the landscape contains only 1 patch, and approaches 1 as the number of different patch types increases, and the proportional distribution of area among patch types becomes more equitable. Overall, seven landscape-related variables were tested. Landscape-related variables may change the velocity of BT spread through their influence on *Culicoides* abundance and species diversity as well as on the probability of contact between hosts and vectors.

Meteorological-related variables. Both the BTV transmission cycle and the lifecycle of its *Culicoides* vectors are affected by temperature and humidity [18,45]. Short term meteorological conditions rapidly can change the age structure and density of *Culicoides* populations, thus modifying the number of midges that can transmit the virus, and consequently the rate of disease transmission [46]. Meteorological conditions also affect the daily flight activity of *Culicoides* vectors [47]. We thus were interested in capturing the

meteorological conditions around the period at which the first animal became infected in a municipality. To account for the uncertainty of the date of the infectious bite, we investigated the effect of temperature and rainfall up to two months prior to the date of the first clinical case reported in each municipality. We consequently considered the monthly average of maximal daily temperatures and monthly total rainfall one month and two months before the first case of BTV-8. These meteorological covariates are referred to as one month-lag and two month-lag, respectively.

To prevent multicollinearity, we avoided including highly correlated covariates simultaneously in a model. To test for correlations among all the candidate variables we used Spearman's rank correlation ρ because the covariates were not normally distributed. This statistics is the most commonly used non-parametric test for correlation; as commonly done, we used a threshold of |0.80|. The covariates were not highly correlated and could be included simultaneously in a model.

To identify non-monotonic and nonlinear responses and determine whether the environmental covariates should be considered as continuous or categorical, we examined the linearity of the relation between each continuous covariate and the response variable. Based on biological relevance, each continuous covariate was discretized: it was divided into four classes of approximately similar size, with the exception of the vaccination covariate which was divided into two classes due to a large number of municipalities with no animals vaccinated at the date of the first clinical case. The response variable, *i.e.*, the velocity of BT spread, was then plotted against each discretized categorical variable, and we visually examined each graph to detect nonlinear variations. If the variation was approximately linear between each class, we used the continuous covariate as a candidate variable in the model;

otherwise, we used the categorical covariate. Finally, we obtained 4 continuous and 12 categorical candidate variables. Their characteristics are summarized in Table 1.
