# Supplementary material for: Did Vaccination Slow the Spread of Bluetongue in France?
Source: PLoS One. 2014 Jan 21;9(1):e85444. doi: 10.1371/journal.pone.0085444 (PMC3897431; doi:10.1371/journal.pone.0085444)
Supplement: Material S4 — Description of the residuals autocovariate (RAC) model used to study the effect of environmental factors on the velocity of BT spread. (PDF) [file pone.0085444.s010.pdf]

**Supplementary material S4: description of the residuals autocovariate (RAC) model used to study the effect of environmental factors on the velocity of BT spread**

The analysis required three steps.

We first applied standard linear regression methods based on ordinary least squares (OLS) on the model building dataset, and fitted a model describing the influence of the covariates on the velocity of BT spread. We used backward model selection based on AICc to select the best model based on both model fit and model complexity [1]. As recommended by Burnham and Anderson [1], we considered that two nested models differing by less than 2 AICc points received identical support from the data. In such a situation, the model with fewer parameters was preferred. We thus obtained an OLS model, but spatial autocorrelation was detected in the model residuals (Supplementary Fig. S2A). We consequently used residuals autocovariate (RAC) models to account for this spatial autocorrelation. A thorough presentation of the RAC model is provided in Crase et al. 2012 [2]. In the RAC model, spatial autocorrelation is accounted for by estimating the strength of the relationship between the OLS residuals and the values of those residuals at neighboring locations. The second step of the analysis was to calculate the autocovariate. To do so, the observations, *i.e.*, the velocity of BT spread, were arranged in a grid and a neighborhood size was defined. We used a raster grid with a 0.9 km resolution to prevent having more than one municipality per raster cell. We used focal calculations with a neighborhood size of 3.6 km. In a preliminary step we tested several neighborhood sizes ranging from 1.8 to 9 km and finally selected a neighborhood size of 3.6 km, which accounted for most of the spatial autocorrelation while including enough municipalities as neighbor. The autocovariate was calculated as the average of the residuals of the best OLS model within the neighborhood size of 3.6 km. In the third step the

environmental covariates and the residuals autocovariate were fitted to a linear regression and the parameter values describing the influence of explanatory variables and the autocovariate on the velocity of BT spread were simultaneously estimated.


1. Burnham KP, Anderson DR (2002) Model selection and Multi-Model Inference, a practical information-theoretic approach. New-York: Springer-Verlag. 488 p.
2. Crase B, Liedloff AC, Wintle BA (2012) A new method for dealing with residual spatial autocorrelation in species distribution models. Ecography 35: 879-888.
